# Exploratory Biomarker-Defined Phenotypic Heterogeneity Beyond a Single Physiological Reserve Construct

**DOI:** 10.3390/jpm16090481

**Published:** 2026-09-18

**Authors:** Muneera O. AlTaweel, Aftab Ahmed Jalbani, Fahad Memon, Ayman Aly Mahmoud, Paras Memon, Islam Elnasharty, Amin Elshehawey, Ali Al Qarni

**Affiliations:** 1 Department of Cardiac Sciences, King Abdulaziz Hospital, MNGHA, King Abdullah Military City, Al-Mubarraz 36248, Al-Ahsa, Saudi Arabiamemonfa@mngha.med.sa (F.M.); mahmouday@mngha.med.sa (A.A.M.); memonpa@mngha.med.sa (P.M.);; 2 King Abdullah International Medical Research Center—Eastern Region (KAIMRC-ER), King Abdullah Military City, Al-Mubarraz 36428, Al-Ahsa, Saudi Arabia; 3 King Saud Bin Abdulaziz University for Health Sciences (KSAU-HS), MNGHA, King Abdullah Military City, Al-Mubarraz 36428, Al-Ahsa, Saudi Arabia; 4 Endocrinology and Metabolism, Department of Medicine, King Abdulaziz Hospital, MNGHA, King Abdullah Military City, Al-Mubarraz 36428, Al-Ahsa, Saudi Arabia

**Keywords:** personalized medicine, biomarker-defined phenotyping, patient heterogeneity, model-based clustering, cardiorenal syndrome, physiological reserve, Gaussian mixture modeling

## Abstract

**Background**: Patient heterogeneity in routine biomarkers is often collapsed into a single physiological reserve score, but whether that one-dimensional representation holds across acute and chronic settings—or whether it obscures biomarker-defined subgroups relevant to individualized care—is unclear. **Methods**: We harmonized routine biomarkers (BMI, blood pressure, eGFR, LDL, total cholesterol, and HbA1c) across two cohorts from the same health system: an acute cardiorenal syndrome cohort and a chronic outpatient cohort treated with dapagliflozin and/or semaglutide, excluding 15 patients common to both. We tested whether these biomarkers supported a one-factor structure via exploratory factor analysis, then a categorical alternative via Gaussian mixture modeling, assessed six-month class persistence in the chronic cohort, and applied the derived classes to the acute cohort for descriptive independent cohort application. **Results**: A one-factor representation was not supported (KMO = 0.485; covariance concentrated in two definitionally related variable pairs). Gaussian mixture modeling favored a two-class solution, distinguished mainly by LDL, total cholesterol, HbA1c, and diastolic blood pressure. Class assignment showed moderate six-month agreement (κ = 0.46) in a subgroup enriched for closer monitoring. In the acute cohort, class structure was not clearly associated with age, BNP, ejection fraction, length of stay, CKD stage, HF phenotype, NYHA class, or comorbidities; associations with outcomes were descriptive, not causal. **Conclusions**: Rather than reducing to a single reserve score, these biomarkers were better described by an exploratory two-class, biomarker-defined phenotype structure that captured observable patient heterogeneity, showed moderate short-term persistence, and was not clearly related to acute illness severity. These findings are specific to the biomarkers and cohorts studied and should not be generalized without independent replication.

## 1. Introduction

Personalized approaches to cardiometabolic risk depend on recognizing that patients who look similar on aggregate scores can differ meaningfully in their underlying biomarker profiles—heterogeneity that a single composite score is, by construction, unable to capture. Physiological reserve—the capacity of a system to withstand and recover from stress—is a foundational concept across aging biology and clinical medicine, and has often been operationalized through composite measures that collapse multiple biomarkers into a single derived score. In geriatric physiology, this depletion of reserve with age has been termed homeostenosis: organ systems increasingly rely on their remaining functional capacity simply to maintain homeostasis at rest, leaving less available to respond to an added challenge [1]. Fried and colleagues operationalized a related idea clinically, describing frailty as a phenotype arising from depleted physiological reserve across multiple interacting systems, distinct from either comorbidity or disability alone [2]. In cardiorenal medicine specifically, the concept of reserve underlies the bidirectional cardiorenal syndrome framework, in which acute or chronic dysfunction in one organ (heart or kidney) can precipitate dysfunction in the other—a formulation that implicitly treats each organ’s reserve as a shared, interacting resource rather than an independent property [3,4,5]. This bidirectional organ crosstalk has more recently been situated within the broader cardiovascular–kidney–metabolic (CKM) health framework, which conceptualizes cardiac, renal, and metabolic dysfunction as an interconnected continuum rather than as isolated organ-specific processes [6,7]. More broadly, reserve has also been conceptualized as a dynamic response revealed only under physiological stress, rather than a static characteristic measurable at rest [1].

A common but rarely tested assumption underlying this framework is that physiological reserve can be represented as a single continuous latent construct, reflected in the correlated behavior of biomarkers spanning renal, hemodynamic, and metabolic domains and ultimately summarized by a composite score. This chain—from reserve as a biological property, to a latent construct inferred statistically from biomarker covariance, to a quantitative score derived from that construct—is rarely made explicit in the literature, and each step carries its own assumptions that need not hold together. This assumption has intuitive appeal—it would allow a single derived score to stand in for an otherwise diffuse, multi-system concept—but it has not, to our knowledge, been directly tested against biomarker data spanning both acute and chronic disease states.

This study tested that assumption directly against an alternative. Under Hypothesis A, physiological reserve exists as one continuous latent construct, identifiable from correlations among routine biomarkers regardless of clinical context. Any “reserve score” discussed below refers to a quantitative summary that would be derived from this construct within our own data, not a pre-existing validated instrument. Under the alternative hypothesis, cross-sectional physiology is instead heterogeneous and phenotype-based: biomarkers cluster into a small number of discrete, biologically coherent groups rather than collapsing onto a single axis.

We tested these hypotheses using biomarker data spanning two clinically distinct populations from the same health system—an acute cardiorenal syndrome cohort at the point of decompensation, and a chronic outpatient cohort undergoing pharmacologic cardiometabolic risk modification—under the hypothesis that, if physiological reserve is indeed a single global construct, it should be detectable across both acute and chronic physiological states—while recognizing that differing biological processes and temporal dynamics across these contexts could equally argue against such invariance. This approach parallels a growing body of recent work applying unsupervised clustering to routine biomarker panels to uncover clinically meaningful phenotypes beyond single composite risk scores, in populations ranging from population-based cardiometabolic cohorts to acute coronary syndromes [8,9].

## 2. Methods

### 2.1. Data Sources and Harmonization

Two datasets from the same health system were harmonized with respect to seven shared biomarkers: (1) a cardiorenal syndrome (CRS) cohort of patients admitted with acute decompensated heart failure, using index admission values, and (2) a chronic outpatient cohort treated with dapagliflozin and/or semaglutide [10,11], using baseline (pre-treatment) values. Seven biomarkers were common to both datasets and retained for analysis: body mass index (BMI), systolic and diastolic blood pressure (SBP, DBP), estimated glomerular filtration rate (eGFR), LDL cholesterol, total cholesterol, and HbA1c. As detailed in Section 2.2, Phase I pooled the standardized data from both harmonized cohorts to test for a single shared construct, while later phases used the two cohorts separately (chronic cohort for class derivation and persistence, CRS cohort for independent cohort application). B-type natriuretic peptide (BNP) was available in both cohorts but excluded from construct building because it is not routinely ordered in stable outpatients, producing non-random (indication-driven) missingness in the chronic cohort; BNP was retained separately as a clinical covariate and marker of acute illness severity, compared descriptively between classes (Section 2.3) rather than entered into an adjusted multivariable model. Free-text and placeholder values (e.g., “not done,” embedded dates, comma–decimal entries) were parsed and validated against physiologically plausible ranges before analysis. Fifteen patients with medical record numbers appearing in both source datasets were identified and excluded from both cohorts to preserve between-group independence.

Biomarkers were selected primarily for their availability in both source datasets, and secondarily for their conventional association with the physiological domains most frequently invoked in reserve and cardiorenal syndrome frameworks: hemodynamic status (blood pressure), renal function (eGFR), and metabolic status (BMI, lipids, HbA1c). This selection prioritized cross-cohort availability over a pre-specified theoretical model of reserve, and the resulting construct validity is accordingly limited: the absence of a single latent factor may reflect a true absence of shared covariance among these particular biomarkers, an incomplete sampling of the physiological domains relevant to reserve, or both. Biomarkers more specific to hemodynamic or renal reserve—for example, natriuretic peptides at scale, cystatin C, or exercise- or stress-based functional measures, several of which have been proposed as components of contemporary multi-marker panels for acute cardiorenal syndrome [12]—were not available in both cohorts and could not be included; their absence should be considered when interpreting the Phase I result.

For the chronic outpatient cohort (dapagliflozin/semaglutide), data were collected retrospectively from 1 December 2020, to 2 May 2024, under IRB approval number 00000299226 (study NRA26/004/2). For the acute cardiorenal syndrome (CRS) cohort, data were collected retrospectively from 1 January 2019, to 30 May 2024, under IRB approval number 00000299626 (study NRA26/006/2).

### 2.2. Construct Building (Phases I–III)

This study used a four-phase analytic architecture spanning three analysis samples. Phase I dimensionality screening (testing whether a single reserve construct is detectable across both acute and chronic physiological states) was performed on the pooled, within-cohort-standardized sample from both cohorts (N = 571). Phases II and III (class derivation and six-month persistence, described below) used only the chronic outpatient cohort’s baseline (pre-treatment) data (N = 420), to avoid conflating the derived class structure with treatment response or cross-cohort case-mix differences. Phase IV (Section 2.3) applied the chronic-derived model, without refitting, to the acute CRS cohort (N = 151). Sample adequacy was assessed with the Kaiser–Meyer–Olkin (KMO) measure [13] and Bartlett’s test of sphericity [14]. Latent dimensionality was assessed via eigenvalue decomposition of the correlation matrix followed by exploratory factor analysis (EFA; minimum residual (minres) extraction, Kaiser criterion for factor retention, reported in Results 3.1). A categorical alternative to the one-factor model was tested using Gaussian mixture modeling (GMM) across one to ten classes, with model selection by Bayesian Information Criterion (BIC) [15], comparing diagonal and full covariance structures [16], an approach consistent with recent applications of finite Gaussian mixture modeling to distinguish cardiometabolic biomarker-defined subgroups [17]. All biomarkers were standardized (z-scored) within cohort prior to modeling. Six-month persistence of class assignment was assessed in the subset of chronic cohort patients with complete data at both baseline and follow-up, by comparing each patient’s baseline-derived class label with their follow-up class label (assigned using the baseline standardization applied to follow-up values), and quantifying baseline-to-follow-up agreement with Cohen’s kappa [18].

### 2.3. Application to a Separate Cohort Without Refitting (Phase IV)

The two-class Gaussian mixture model derived in Section 2.2 (chronic cohort baseline, N = 420) was applied, without refitting, to the acute CRS cohort (index admission, N = 151): each CRS patient’s seven biomarkers were standardized using the chronic cohort’s baseline standardization parameters, and posterior class probabilities from the existing model were used to assign each patient to Class 1 or Class 2. No new model was fit in the CRS cohort; this step evaluates whether the chronic cohort-derived classification can be applied, without refitting, to a clinically distinct independent sample, not whether the same structure is independently recoverable in both cohorts. Class outcome associations were tested against clean, pre-derived binary outcome fields (in-hospital death, rehospitalization at 2 and 6 months, dialysis need at 2 months, prolonged length of stay [>75th percentile], and a composite adverse in-hospital event) using Fisher’s exact test. To evaluate whether class assignment reflected acute illness severity rather than a distinct construct, the classes’ clinical characteristics and markers of acute illness severity were compared on age, BNP, ejection fraction, length of stay, sex, CKD stage, heart failure phenotype, NYHA class, and comorbidity burden (diabetes, hypertension, coronary artery disease, cerebrovascular disease, chronic heart failure, peripheral vascular disease, prior acute kidney injury), using Mann–Whitney U tests for continuous variables and chi-square tests for categorical variables.

### 2.4. Sensitivity and Stability Analyses

We assessed two potential threats to the robustness of the two-class solution. First, because LDL and total cholesterol were highly correlated (r = 0.90 in the pooled Phase I sample and r = 0.93 in the chronic derivation cohort) and jointly drove class separation, we refit the Gaussian mixture model on the remaining six biomarkers after dropping each variable in turn. Dropping total cholesterol preserved a two-class BIC minimum and produced classes in substantial agreement with the original seven-biomarker solution (kappa = 0.69, best-aligned). Dropping LDL weakened but did not eliminate the two-class signal: BIC no longer favored two classes over one, though a forced two-class solution still agreed moderately with the original classes (kappa = 0.63). The two-class structure is therefore only partly independent of the LDL/total cholesterol pair and should be interpreted as biomarker panel-dependent rather than fully robust to the removal of either lipid variable.

Second, we assessed the stability of the two-class solution using 500 bootstrap resamples of the chronic cohort baseline data (N = 420, resampled with replacement). The Gaussian mixture model was refit on each resample across one to five classes; a two-or-more-class solution was BIC-preferred in 92.2% of resamples and a two-class solution specifically in 63.8%. To assess agreement independent of each resample’s own BIC-preferred class count, a two-class model was additionally forced on every resample regardless of its BIC-preferred solution, and the resulting class labels were scored back onto the original sample; this unconditional approach gave a mean adjusted Rand index of 0.57 (95% range 0.04–0.80) against the original class assignment, materially unchanged whether or not that resample’s own BIC preferred two classes. The minority class proportion was stable across resamples (median 30.2%, versus 29.8% in the original solution). This unconditional bootstrap strategy—forcing the target class count on every resample rather than only on resamples whose own model selection criterion favored it—follows recent methodological work on assessing the sampling uncertainty robustness of cluster-derived typologies [19]. Together with the LDL/total cholesterol sensitivity analysis, these results indicate that the two-class structure is a moderately, not uniformly, stable feature of this biomarker panel, consistent with its treatment throughout as an exploratory rather than confirmed phenotype structure.

We considered a categorical two-class model rather than a continuous multidimensional alternative because the one-factor model’s failure in Phase I (Section 3.1) reflected an absence of shared covariance across most of the panel, not evidence for an alternative continuous structure; a multidimensional continuous model would still require specifying how many dimensions to retain, and the same eigenvalue and communality pattern that ruled out one factor did not support a clear multi-factor alternative. The categorical approach was adopted because it directly tested whether biomarkers instead covary differently within subgroups, and because it yielded testable predictions (class persistence, out-of-sample application) that a continuous latent dimension model would not straightforwardly provide with this panel.

## 3. Results

Baseline characteristics of both cohorts are summarized in Table 1.

**Table 1:** Cohort characteristics (complete-case, construct variables).

Characteristic	CRS (Acute, Index Admission)	Dapa/Sema (Chronic, Baseline)
N	151	420
Sex, n (%)	Male 80 (53.0%), Female 71 (47.0%)	Female 221 (52.6%), Male 198 (47.1%), Missing 1 (0.2%)
Age	Mean 68.3 years (median 68.0)	Reported in banded categories; modal bands 51–60 and 61–70 years
Treatment exposure	N/A (acute admission)	Dapagliflozin 339 (80.7%); Semaglutide 81 (19.3%)

CRS: cardiorenal syndrome.

### 3.1. Phase I: A Single Latent Factor Was Not Supported

Sample adequacy for factor analysis was below the conventional threshold for factorability (KMO = 0.485; Bartlett’s χ^2^ = 1147.5, *p* < 0.001, though uninformative at this sample size). The correlation matrix (Table 2) showed meaningful covariance only between two variable pairs related by measurement or definition—systolic and diastolic blood pressure (r = 0.40) and LDL and total cholesterol (r = 0.90, the latter reflecting that LDL is a computed component of total cholesterol). BMI, eGFR, and HbA1c showed near-zero correlation with each other and with both pairs (|r| generally < 0.12). A three-factor exploratory factor analysis (minimum residual (minres) extraction with varimax rotation; three factors retained by the Kaiser criterion, eigenvalues 1.96, 1.45, and 1.08) confirmed this: only the blood pressure pair and the lipid pair loaded meaningfully (communalities 0.59–0.996), while BMI, eGFR, and HbA1c had communalities of 0.03–0.05, indicating these variables share almost no variance with any extracted factor.

### 3.2. Phase II: A Two-Class Phenotype Structure

Gaussian mixture modeling with full covariance produced a clear BIC minimum at two classes (BIC = 7569.7), rising monotonically for three or more classes (BIC = 7678.3 at k = 3, 8080.2 at k = 6). Class 1 (n = 295; lower lipid–glycemic–diastolic blood pressure profile) was characterized by lower LDL, total cholesterol, HbA1c, and diastolic blood pressure relative to Class 2 (n = 125; higher lipid–glycemic–diastolic blood pressure profile); BMI, systolic blood pressure, and eGFR did not distinguish the classes (Table 3). Mean posterior assignment probability was 0.89, indicating reasonably confident class separation. Semaglutide-treated patients were modestly overrepresented in Class 2 (26.4% vs. 16.3%), consistent with semaglutide being added preferentially in harder-to-control patients (confounding by indication) rather than a treatment effect on class membership.

### 3.3. Phase III: Classes Are Moderately Persistent over Six Months

Among 295 patients with complete data at both baseline and 6-month follow-up, class assignment agreement exceeded chance (Cohen’s κ = 0.46; χ^2^ = 61.7, *p* < 0.001). Patients beginning in Class 2 rarely transitioned to Class 1 (12.0%, 95% CI 7.6–16.4%); patients beginning in Class 1 transitioned to Class 2 more often, though this estimate was less precise (43.7%, 95% CI 33.3–54.1%) (Table 4). This paired subsample was itself enriched for baseline Class 2 (70.5% vs. 30% in the full baseline sample), consistent with more frequent follow-up laboratory testing in Class 2 patients; the findings should be interpreted as applying to a monitored, not necessarily representative, subgroup.

### 3.4. Phase IV: Classes Were Not Associated with Acute Illness Severity

Applying the chronic cohort-derived class model to the acute CRS cohort (n = 151) assigned 95 patients (62.9%) to Class 1 and 56 (37.1%) to Class 2,with a similar lipid–glycemic–diastolic pattern observed descriptively in the separate acute cohort, consistent with the Table 3 class profiles). Class 2 was associated with numerically lower rates of adverse outcomes across all measures tested except rehospitalization: in-hospital death (4.9% vs. 9.7%), dialysis need at 2 months (6.5% vs. 9.1%), prolonged length of stay (14.8% vs. 28.7%, *p* = 0.055), and a composite adverse in-hospital event (16.1% vs. 32.7%, *p* = 0.028) (Table 5). Six-month rehospitalization data were available for a smaller subset (n = 66) and showed no meaningful difference between classes (22.2% vs. 23.1%, OR = 1.05, *p* = 1.000), consistent with the null 2-month rehospitalization finding.

Comparison of the two classes on variables directly relevant to acute severity showed no meaningful separation: age, BNP, ejection fraction, length of stay, sex, CKD stage, heart failure phenotype, NYHA class, and comorbidity burden (diabetes, hypertension, coronary artery disease, cerebrovascular disease, chronic heart failure, peripheral vascular disease, prior acute kidney injury) all showed *p* > 0.09 (Table 6). These outcome associations are drawn from a single independent acute cohort and are reported here as associative rather than causal; no causal or mechanistic interpretation is warranted without replication.

### 3.5. Sensitivity and Stability of the Two-Class Solution

Dropping total cholesterol from the six remaining biomarkers preserved a two-class BIC minimum and reproduced classes in substantial agreement with the original solution (kappa = 0.69). Dropping LDL weakened the categorical signal: BIC no longer favored two classes over one, though a forced two-class solution still showed moderate agreement with the original classes (kappa = 0.63)—indicating that lipid information, and LDL in particular, contributes materially to the two-class structure rather than being incidental to it. Across 500 bootstrap resamples of the chronic cohort baseline data, a two-or-more-class solution was BIC-preferred in 92.2% of resamples (two-class specifically in 63.8%). Forcing a two-class fit on every resample regardless of its own BIC-preferred solution—to avoid conditioning agreement on resamples that happened to favor two classes—gave a mean adjusted Rand index of 0.57 (95% range 0.04–0.80) against the original assignment. The minority class proportion was stable across resamples (median 30.2% vs. 29.8% in the original solution). These results provide moderate but incomplete evidence of stability: agreement with the original classification was consistently positive but variable across resamples (ARI range 0.04–0.80), indicating the two-class structure is only partly dependent on the correlated LDL/total cholesterol pair rather than either fully robust or purely an artifact of that collinearity.

## 4. Discussion

**Figure 1:**
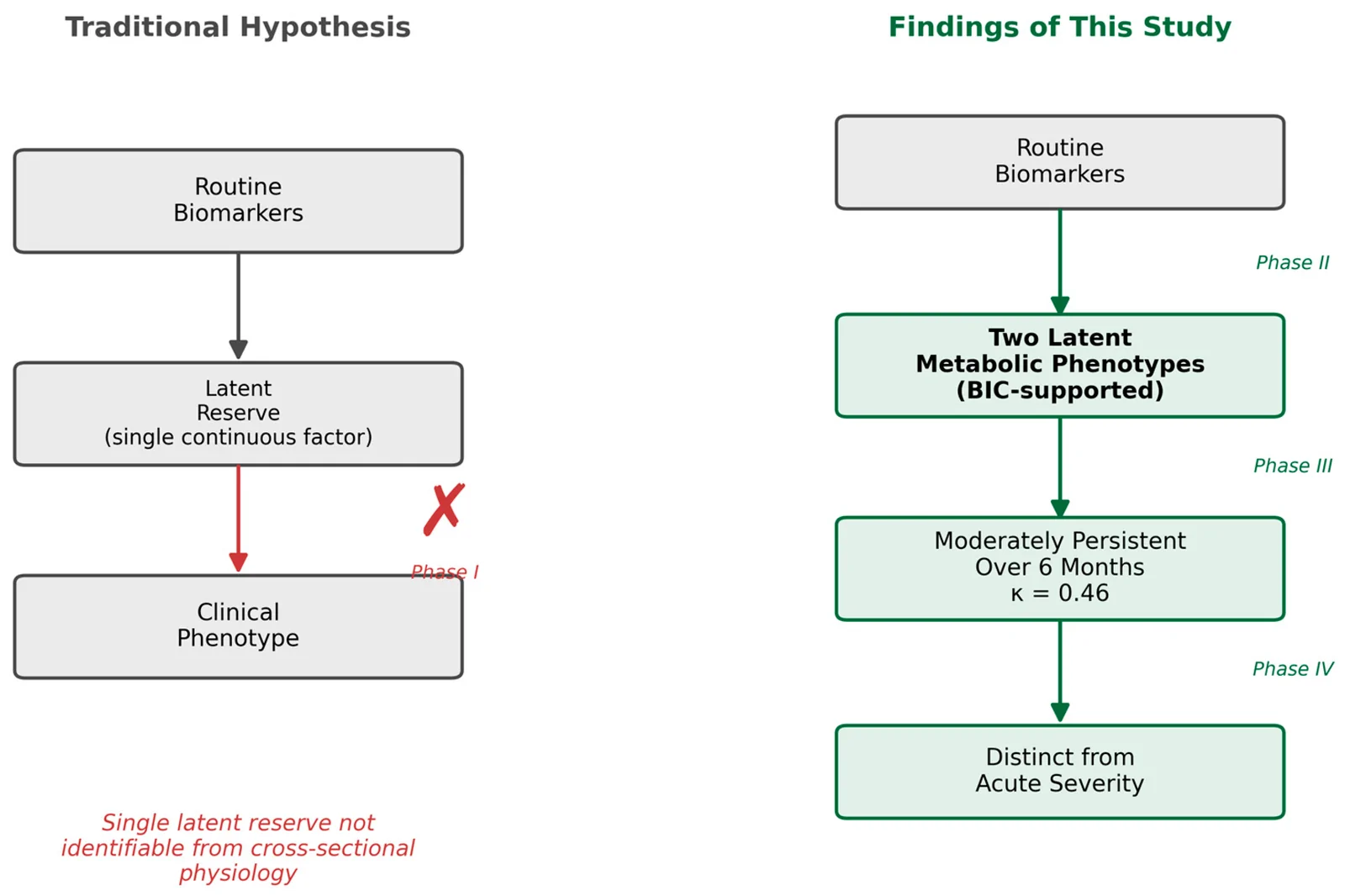
Conceptual framework of the study. The traditional model assumes that routine biomarkers reflect a single continuous latent physiological reserve that determines clinical phenotype. In contrast, the present study found that cross-sectional physiology did not support a one-factor biomarker representation of reserve in these cohorts, but instead identified a two-class phenotype structure that showed moderate persistence over six months and was not associated with acute illness severity when applied to an independent acute cohort.

Figure 1 summarizes the conceptual framework tested in this study and the resulting findings.

This study tested whether this biomarker panel—routine renal, hemodynamic, and metabolic measures collected cross-sectionally—supports a single one-factor representation of physiological reserve. The biomarker panel did not support a coherent one-factor representation of physiological reserve. Although Bartlett’s test indicated that the correlation matrix differed from an identity matrix, the low KMO value indicated limited sampling adequacy for factor analysis, and the observed covariance was concentrated primarily in two related variable pairs. Instead, the data were more consistent with a two-class biomarker-defined phenotype structure identified through Gaussian mixture modeling. This structure showed moderate persistence over six months and was not clearly associated with acute illness severity when examined in an independent CRS cohort.

Rather, the observed correlation structure did not exhibit the broad shared covariance expected under a single latent factor. Shared variance was confined to two variable pairs related by measurement or definition (systolic and diastolic blood pressure; LDL and total cholesterol), while renal function, glycemic control, and adiposity varied largely independently of one another and of either pair.

This does not mean the panel has no structure at all—only that the structure is not one-dimensional. A mixture model does not require indicators to covary across an entire sample; it requires only that they covary differently within subgroups, which is what the two-class solution captured. Failure to identify a one-factor reserve representation should not be read as failure to identify any organization in the data; rather, it suggests that this biomarker panel is organized through heterogeneous, biomarker-defined phenotypes rather than a single continuous axis. This distinction reflects phenotype discovery rather than confirmation of a fixed biological structure, and should be treated as a candidate finding pending replication in additional cohorts.

Whether these classes are biologically meaningful, rather than an artifact of the modeling approach, is best judged from their behavior over time. The moderate six-month persistence of class assignment (κ = 0.46) is more consistent with a non-arbitrary partition than with a modeling artifact, though this observation is preliminary, drawn from a single cohort followed for six months, and the evidence for a biologically meaningful class structure remains modest given the small number of indicators, their partial definitional linkage, and the enrichment of the follow-up subgroup for closer clinical monitoring.

What these classes represent biologically is best described as a biomarker-defined distinction rather than a marker of acute illness severity. When applied to a separate acute cardiorenal syndrome cohort, the classes did not track age, BNP, ejection fraction, length of stay, or comorbidity burden—yet Class 2 was associated with numerically fewer adverse in-hospital events. This dissociation was not anticipated and was not directly tested for an underlying mechanism; drawn from a single separate acute cohort, it should be treated as hypothesis-generating rather than an established or causal finding. A biomarker-defined class that is dissociated from conventional severity markers is not unique to this cohort: recent unsupervised clustering studies in acute cardiac populations have similarly identified subphenotypes whose outcome differences were not explained by standard severity indices alone [20,21,22].

Taken together, these results suggest that this biomarker panel, examined cross-sectionally, is better suited to exploratory phenotype discovery than to estimating a single quantitative reserve score. The two-class structure was internally consistent across construct building, persistence, and separate cohort application, whereas a one-factor representation was not, at any stage of the analysis. For personalized care, this distinction matters practically: a biomarker-defined phenotype label carries information about a patient’s metabolic profile and its short-term stability that a single collapsed reserve score would discard, and could in principle support differentiated monitoring intensity once independently validated. This pattern should be regarded as a discovered candidate phenotype structure rather than an established biological taxonomy until independently replicated.

Future work should determine whether dynamic physiological perturbation or serial measurements reveal a reserve-related structure that this cross-sectional biomarker panel could not, a direction consistent with recent conceptual work distinguishing static reserve from the dynamic, recovery-oriented construct of physiological resilience [23].

## 5. Methodological Boundary of Cross-Sectional Design

Within the limits of the routinely collected, single-timepoint biomarkers used in this study, a single latent reserve construct was not identifiable, and this negative Phase I finding is itself a substantive result of that specific test. This study cannot evaluate whether dynamic reserve mechanisms—temporal adaptation, response to physiological perturbation, or nonlinear threshold behavior—would be identifiable using a different measurement approach, since these properties, by definition, require observing a system under stress rather than at rest. Testing reserve as a dynamic property directly would require serial measurement through a defined physiological perturbation, a design outside the scope of the present cross-sectional study.

## 6. Limitations

• The KMO measure of sampling adequacy was below conventional thresholds (KMO = 0.485), indicating limited suitability of this biomarker correlation structure for factor analysis; this reflects the factorability of the observed correlations among these seven biomarkers, not an inadequate sample size, and should be interpreted accordingly.
• Both cohorts were drawn from a single health system, limiting generalizability of the identified phenotype structure to other populations and settings.
• The six-month persistence analysis relied on a paired subsample enriched for patients selected for closer clinical monitoring (70.5% baseline Class 2 vs. 30% in the full baseline sample), so persistence estimates apply to a monitored subgroup rather than the general treated population.
• Modest sample size in the acute CRS cohort (n = 151 for construct-relevant complete cases), limiting precision for some outcome comparisons (e.g., 6-month rehospitalization, n = 66).
• Residual and unmeasured confounding cannot be excluded, particularly confounding by indication in the association between semaglutide exposure and class membership, given semaglutide’s own established, independent effect on cardiovascular risk [24].
• BNP missingness in the chronic outpatient cohort was informative (associated with clinical suspicion) rather than random, which motivated its exclusion from construct building but limits its use elsewhere in the analysis.
• Follow-up was limited to six months; longer-term class persistence and its relationship to hard clinical outcomes remains untested.

## 7. Conclusions

Rather than collapsing onto a single physiological reserve score, the biomarker structure identified in the chronic outpatient cohort revealed exploratory biomarker-defined heterogeneity, better described by a two-class phenotype driven mainly by lipid–glycemic and diastolic blood pressure patterns; this structure showed moderate six-month persistence in a clinically monitored subgroup and was successfully applied, without refitting, to an independent acute cardiorenal syndrome cohort.

These findings should be interpreted cautiously. The factor analysis result was limited by low sample adequacy, the biomarker panel was incomplete with respect to reserve biology, and the separate cohort application was descriptive rather than causal. Within the biomarker panel studied, routine cross-sectional measurements appeared more informative for exploratory phenotype discovery than for constructing a single quantitative biomarker representation of physiological reserve.

## Figures and Tables

**Table 2:** Correlation matrix of shared biomarkers, within-cohort standardized, pooled across both cohorts (N = 571).

	BMI	SBP	DBP	eGFR	LDL	Total Chol.	HbA1c
BMI	1.00	0.21	0.02	−0.00	0.03	0.04	0.01
SBP	0.21	1.00	0.40	−0.07	−0.01	0.06	0.12
DBP	0.02	0.40	1.00	0.06	0.09	0.08	0.02
eGFR	−0.00	−0.07	0.06	1.00	0.06	0.03	−0.07
LDL	0.03	−0.01	0.09	0.06	1.00	0.90	0.06
Total Chol.	0.04	0.06	0.08	0.03	0.90	1.00	0.10
HbA1c	0.01	0.12	0.02	−0.07	0.06	0.10	1.00

BMI: body mass index; SBP: systolic blood pressure; DBP: diastolic blood pressure; eGFR: estimated glomerular filtration rate; LDL: low-density lipoprotein; HbA1c: glycated haemoglobin.

**Table 3:** Two-class Gaussian mixture model profile (Dapa/Sema baseline, N = 420).

Variable	Class 1 (n = 295)	Class 2 (n = 125)
LDL (mmol/L)	2.16	3.25
Total cholesterol (mmol/L)	3.67	5.05
HbA1c (%)	8.26	9.77
Diastolic BP (mmHg)	69.1	77.3
BMI (kg/m^2^)	34.7	33.2
Systolic BP (mmHg)	136.7	138.4
eGFR (mL/min/1.73 m^2^)	79.4	80.3

LDL: low-density lipoprotein; HbA1c: glycated haemoglobin; BP: blood pressure; BMI: body mass index; eGFR: estimated glomerular filtration rate.

**Table 4:** Six-month class transition matrix (N = 295 paired patients).

Baseline Class	Remained Same Class at 6 Months	Transitioned to Other Class
Class 1 (n = 87)	56.3%	43.7% (95% CI 33.3–54.1%)
Class 2 (n = 208)	88.0%	12.0% (95% CI 7.6–16.4%)

Note: The N = 295 paired subsample in this table is distinct from the N = 420 full baseline cohort in Table 3; it reflects patients with complete data at both baseline and six-month follow-up and is enriched for baseline Class 2, as described in the text.

**Table 5:** Clinical outcomes by class, CRS cohort (acute, index admission).

Outcome	Class 1	Class 2	OR	*p*
In-hospital death	9.7%	4.9%	0.48	0.375
Dialysis need (2 mo)	9.1%	6.5%	0.69	0.768
Prolonged LOS (>P75)	28.7%	14.8%	0.43	0.055
Composite adverse in-hospital event	32.7%	16.1%	0.40	0.028
Rehospitalization (2 mo)	27.7%	31.8%	1.22	0.673
Rehospitalization (6 mo, n = 66)	23.1%	22.2%	1.05	1.000

OR: odds ratio; LOS: length of stay.

**Table 6:** Clinical characteristics and markers of acute illness severity by biomarker-defined class, CRS cohort.

Variable	Class 1	Class 2	*p*
Age, median (IQR)	68.5 (62.2–76.0)	65.0 (60.0–76.0)	0.253
BNP, median (IQR)	201.0 (94.0–497.0)	210.0 (81.0–364.0)	0.758
EF%, median (IQR)	43.8 (25.0–57.5)	37.5 (25.0–54.4)	0.281
Length of stay, median (IQR)	5.0 (3.0–8.0)	4.0 (3.0–6.0)	0.093
Sex distribution	—	—	0.518
CKD stage distribution	—	—	0.465
HF phenotype distribution	—	—	0.188
NYHA class distribution	—	—	0.743
Diabetes, hypertension, CAD, CVA, CHF, PVD, prior AKI	—	—	all *p* > 0.48

IQR: interquartile range; BNP: B-type natriuretic peptide; EF: ejection fraction; CKD: chronic kidney disease; HF: heart failure; NYHA: New York Heart Association; CAD: coronary artery disease; CVA: cerebrovascular accident; CHF: chronic heart failure; PVD: peripheral vascular disease; AKI: acute kidney injury.

## Data Availability

The data underlying this study contain sensitive patient health information collected under institutional IRB approval and are not publicly available. De-identified data may be made available from the corresponding author upon reasonable request and subject to appropriate institutional and ethics approval.

## References

[1] Taffet G.E., Wasserman M.R., Bakerjian D., Linnebur S. (2024). Physiology of aging. Geriatric Medicine: A Person Centered Evidence Based Approach.

[2] Fried L.P., Tangen C.M., Walston J., Newman A.B., Hirsch C., Gottdiener J., Seeman T., Tracy R., Kop W.J., Burke G. (2001). Frailty in older adults: Evidence for a phenotype. J. Gerontol. A Biol. Sci. Med. Sci..

[3] Ronco C., Haapio M., House A.A., Anavekar N., Bellomo R. (2008). Cardiorenal syndrome. J. Am. Coll. Cardiol..

[4] Stefanou E., Tountas C., Ioannidis E., Kole C. (2024). Biomarkers in cardiorenal syndrome, a potential use in precision medicine. J. Nephrol..

[5] Aletras G., Bachlitzanaki M., Stratinaki M., Lamprogiannakis E., Petrakis I., Foukarakis E., Pantazis Y., Hamilos M., Stylianou K. (2025). Integrating novel biomarkers into clinical practice: A practical framework for diagnosis and management of cardiorenal syndrome. Life.

[6] Ndumele C.E., Rangaswami J., Chow S.L., Neeland I.J., Tuttle K.R., Khan S.S., Coresh J., Mathew R.O., Baker-Smith C.M., Carnethon M.R. (2023). Cardiovascular-Kidney-Metabolic Health: A Presidential Advisory from the American Heart Association. Circulation.

[7] Cai X., Huang H., Li T. (2025). Cardiovascular-kidney-metabolic: Hype or a focus on front-line health?. J. Transl. Intern Med..

[8] Coral D.E., Smit F., Farzaneh A., Gieswinkel A., Tajes J.F., Sparsø T., Delfin C., Bauvin P., Wang K., Temprosa M. (2025). Subclassification of obesity for precision prediction of cardiometabolic diseases. Nat. Med..

[9] Xu W., Zhang X., Er L., Gu L., Zhang Z., Li X., Dong R., Cao G., Wang X. (2026). Unsupervised cardiometabolic phenotyping unmasks residual MACCE risk beyond LDL-C in acute myocardial infarction after revascularization. Front. Cardiovasc. Med..

[10] EMPA-KIDNEY Collaborative Group (2023). Empagliflozin in patients with chronic kidney disease. N. Engl. J. Med..

[11] Chertow G.M., Correa-Rotter R., Vart P., Jongs N., McMurray J.J.V., Rossing P., Langkilde A.M., Sjöström C.D., Toto R.D., Wheeler D.C. (2023). Effects of dapagliflozin in chronic kidney disease, with and without other cardiovascular medications: DAPA-CKD trial. J. Am. Heart Assoc..

[12] Jefferies J.L., Kovesdy C.P., Ronco C. (2023). Contemporary laboratory assessment of acute cardiorenal syndrome for early diagnosis: A call for action. Am. Heart J..

[13] Kaiser H.F. (1974). An index of factorial simplicity. Psychometrika.

[14] Bartlett M.S. (1951). The effect of standardisation on a chi square approximation in factor analysis. Biometrika.

[15] Schwarz G. (1978). Estimating the dimension of a model. Ann. Stat..

[16] Fraley C., Raftery A.E. (2002). Model-based clustering, discriminant analysis, and density estimation. J. Am. Stat. Assoc..

[17] Hossain M.J. (2023). A novel application of finite Gaussian mixture model (GMM) using real and simulated biomarkers of cardiovascular disease to distinguish adolescents with and without obesity. Commun. Stat. Case Stud. Data Anal. Appl..

[18] Cohen J. (1960). A coefficient of agreement for nominal scales. Educ. Psychol. Meas..

[19] Roth L., Studer M., Zuercher E., Peytremann-Bridevaux I. (2024). Robustness assessment of regressions using cluster analysis typologies: A bootstrap procedure with application in state sequence analysis. BMC Med. Res. Methodol..

[20] Jentzer J.C., Reddy Y.N.V., Soussi S., Crespo-Diaz R., Patel P.C., Lawler P.R., Mebazaa A., Dunlay S.M. (2024). Unsupervised machine learning to identify subphenotypes among cardiac intensive care unit patients with heart failure. ESC Heart Fail..

[21] Nucifora G., Muser D., Bradley J., Tsoumani Z., De Angelis G., Caiffa T., Schmitt M., Sinagra G., Miller C. (2025). Unsupervised phenotypic clustering of cardiac MRI data reveals distinct subgroups associated with outcomes in ischemic cardiomyopathy. Int. J. Cardiovasc. Imaging.

[22] Kim J. (2025). Metabotype risk clustering based on metabolic disease biomarkers and its association with metabolic syndrome in Korean adults: Findings from the 2016-2023 Korea National Health and Nutrition Examination Survey (KNHANES). Diseases.

[23] Cosarderelioglu C., Walston J.D., Abadir P.M. (2025). From frailty to resilience: Exploring adaptive capacity and reserve in older adults-a narrative review. Front. Aging.

[24] Lincoff A.M., Brown-Frandsen K., Colhoun H.M., Deanfield J., Emerson S.S., Esbjerg S., Hardt-Lindberg S., Hovingh G.K., Kahn S.E., Kushner R.F. (2023). SELECT Trial Investigators. Semaglutide and cardiovascular outcomes in obesity without diabetes. N. Engl. J. Med..

